# Nicotinium hydrogen sulfate

**DOI:** 10.1107/S1600536809034928

**Published:** 2009-09-05

**Authors:** Li-Zhuang Chen

**Affiliations:** a Ordered Matter Science Research Center, College of Chemistry and Chemical Engineering, Southeast University, Nanjing 210096, People’s Republic of China

## Abstract

The structure of title compound, C_6_H_6_NO_2_^+^·HSO_4_^−^, comprises discrete ions which are inter­conected by N—H⋯O and O—H⋯O hydrogen bonds, leading to a neutral one-dimensional network along [001]. These hydrogen bonds appear to complement the Coulombic inter­action and help to stabilize the structure further.

## Related literature

For simple mol­ecular–ionic crystals containing organic cations and acid radicals (1:1 molar ratio), see: Czupiński *et al.* (2002 ▶); Katrusiak & Szafrański (1999 ▶, 2006 ▶). For the structure of dinicotinium sulfate, see: Athimoolam & Rajaram (2005 ▶).
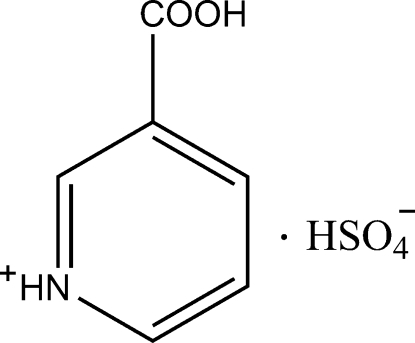


## Experimental

### 

#### Crystal data

C_6_H_6_NO_2_^+^·HSO_4_^−^
*M* *_r_* = 221.19
Monoclinic, 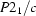

*a* = 8.2654 (17) Å
*b* = 11.545 (2) Å
*c* = 9.4669 (19) Å
β = 109.43 (3)°
*V* = 851.9 (3) Å^3^
*Z* = 4
Mo *K*α radiation
μ = 0.39 mm^−1^
*T* = 293 K
0.25 × 0.2 × 0.2 mm

#### Data collection

Rigaku SCXmini diffractometer
Absorption correction: multi-scan (*CrystalClear*; Rigaku, 2005 ▶) *T*_min_ = 0.91, *T*_max_ = 0.93
8643 measured reflections
1949 independent reflections
1788 reflections with *I* > 2σ(*I*)
*R*_int_ = 0.029

#### Refinement

*R*[*F* ^2^ > 2σ(*F* ^2^)] = 0.034
*wR*(*F* ^2^) = 0.090
*S* = 1.14
1949 reflections
127 parameters
H-atom parameters constrained
Δρ_max_ = 0.21 e Å^−3^
Δρ_min_ = −0.49 e Å^−3^

### 

Data collection: *CrystalClear* (Rigaku, 2005 ▶); cell refinement: *CrystalClear*; data reduction: *CrystalClear*; program(s) used to solve structure: *SHELXS97* (Sheldrick, 2008 ▶); program(s) used to refine structure: *SHELXL97* (Sheldrick, 2008 ▶); molecular graphics: *SHELXTL* (Sheldrick, 2008 ▶); software used to prepare material for publication: *SHELXL97*.

## Supplementary Material

Crystal structure: contains datablocks I, global. DOI: 10.1107/S1600536809034928/bx2233sup1.cif

Structure factors: contains datablocks I. DOI: 10.1107/S1600536809034928/bx2233Isup2.hkl

Additional supplementary materials:  crystallographic information; 3D view; checkCIF report

## Figures and Tables

**Table 1:** Hydrogen-bond geometry (Å, °)

*D*—H⋯*A*	*D*—H	H⋯*A*	*D*⋯*A*	*D*—H⋯*A*
O2—H2*B*⋯O3	0.85	1.83	2.6697 (18)	169
O6—H1⋯O5^i^	0.89	1.73	2.6129 (17)	170
N1—H1*B*⋯O4^ii^	0.86	2.11	2.843 (2)	143

Symmetry codes: (i) 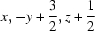; (ii) 
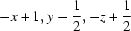
.

**Fig. 1.**
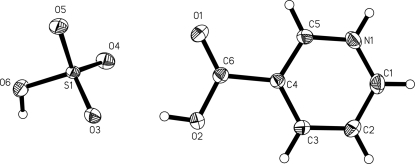
The asymmetric unit of the title compound with atom labels

**Fig. 2.**
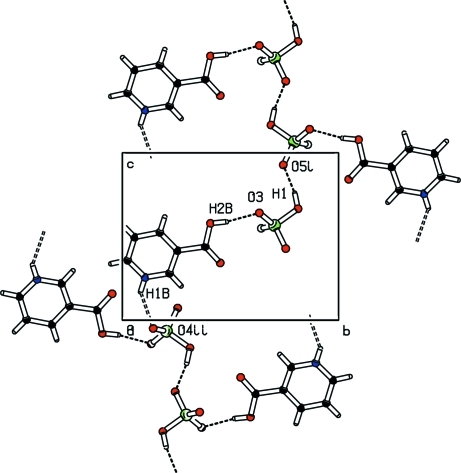
The packing viewed along the a axis. Hydrogen bonds are drawn as dashed lines

## supplementary crystallographic information

### Comment

Recently, much attention has been devoted to simple molecular–ionic crystals containing organic cations and acid radicals (1:1molar ratio) due to the tunability of their special structural features and their interesting physical properties. (*e.g.* Czupiński *et al.*, 2002; Katrusiak & Szafrański, 1999; Katrusiak & Szafrański, 2006) the crystal structure of dinicotinium sulfate compound have been reported (Athimoolam *et al.*, 2005). In our laboratory, a compound containing protoned nicotinic acid and HSO_4_^- ^anions has been synthesized, its crystal structure is reported herein.

The asymmetric unit of the title compound, C_6_H_6_NO_2_^+^.HSO_4_^-^, (Fig.1) consists of protoned nicotinic acid and HSO_4_^- ^anions, the nicotinium cation is essentially planar. The protonation of the N site of the pyridine ring is demonstrated by the C—N bond distances and C—N—C bond angle. Usually, protonation on the aromatic ring leads to a slightly larger C—N—C bond angle (122.9 (2)°). Cations and anions are placed alternately and linked through intermolecular hydrogen bonds (Fig. 2 and Table 1). The structure of title compound C_6_H_6_NO_2_^+^.HSO_4_^-^, comprises discrete ions which are placed alternately and interconected by N—H···O and O—H···O hydrogen bonds leading to a neutral one-dimensional network along [001] direction. These hydrogen bonds appear to complement the Coulombic interaction and help to stabilize the structure further.

### Experimental

Nicotinic acid (10 mmol) and 10% aqueous H_2_SO_4_ in a molar ratio of 1:1 were mixed and dissolved in water by heating to 323 K forming a clear solution. The reaction mixture was cooled slowly to room temperature, crystals of the title compound were formed, collected and washed with dilute aqueous H_2_SO_4_.

### Refinement

All H atoms were placed in calculated positions, with C—H = 0.93 Å, O—H = 0.85 Å and N—H = 0.86 Å, and refined using a riding model, with *U*_iso_(H) = 1.2*U*_eq_(C,*N*) and 1.5*U*_eq_(O).

### Crystal data

**Table:**

C_6_H_6_NO_2_^+^·HSO_4_^−^	*F*(000) = 456
*M**_r_* = 221.19	*D*_x_ = 1.725 Mg m^−^^3^
Monoclinic, *P*2_1_/*c*	Mo *K*α radiation, λ = 0.71073 Å
Hall symbol: -P 2ybc	Cell parameters from 1788 reflections
*a* = 8.2654 (17) Å	θ = 3.2–27.5°
*b* = 11.545 (2) Å	µ = 0.39 mm^−^^1^
*c* = 9.4669 (19) Å	*T* = 293 K
β = 109.43 (3)°	Block, colorless
*V* = 851.9 (3) Å^3^	0.25 × 0.2 × 0.2 mm
*Z* = 4	

### Data collection

**Table:**

Rigaku SCXmini diffractometer	1949 independent reflections
Radiation source: fine-focus sealed tube	1788 reflections with *I* > 2σ(*I*)
graphite	*R*_int_ = 0.029
Detector resolution: 13.6612 pixels mm^-1^	θ_max_ = 27.5°, θ_min_ = 3.2°
ω scans	*h* = −10→10
Absorption correction: multi-scan (*CrystalClear*; Rigaku, 2005)	*k* = −14→14
*T*_min_ = 0.91, *T*_max_ = 0.93	*l* = −12→12
8643 measured reflections	

### Refinement

**Table:**

Refinement on *F*^2^	Primary atom site location: structure-invariant direct methods
Least-squares matrix: full	Secondary atom site location: difference Fourier map
*R*[*F*^2^ > 2σ(*F*^2^)] = 0.034	Hydrogen site location: inferred from neighbouring sites
*wR*(*F*^2^) = 0.090	H-atom parameters constrained
*S* = 1.14	*w* = 1/[σ^2^(*F*_o_^2^) + (0.0405*P*)^2^ + 0.3479*P*] where *P* = (*F*_o_^2^ + 2*F*_c_^2^)/3
1949 reflections	(Δ/σ)_max_ = 0.001
127 parameters	Δρ_max_ = 0.21 e Å^−^^3^
0 restraints	Δρ_min_ = −0.49 e Å^−^^3^

### Special details

**Table:**

Geometry. All e.s.d.'s (except the e.s.d. in the dihedral angle between two l.s. planes) are estimated using the full covariance matrix. The cell e.s.d.'s are taken into account individually in the estimation of e.s.d.'s in distances, angles and torsion angles; correlations between e.s.d.'s in cell parameters are only used when they are defined by crystal symmetry. An approximate (isotropic) treatment of cell e.s.d.'s is used for estimating e.s.d.'s involving l.s. planes.
Refinement. Refinement of *F*^2^ against ALL reflections. The weighted *R*-factor *wR* and goodness of fit *S* are based on *F*^2^, conventional *R*-factors *R* are based on *F*, with *F* set to zero for negative *F*^2^. The threshold expression of *F*^2^ > σ(*F*^2^) is used only for calculating *R*-factors(gt) *etc*. and is not relevant to the choice of reflections for refinement. *R*-factors based on *F*^2^ are statistically about twice as large as those based on *F*, and *R*- factors based on ALL data will be even larger.

### Fractional atomic coordinates and isotropic or equivalent isotropic displacement parameters (Å^2^)

**Table:**

	*x*	*y*	*z*	*U*_iso_*/*U*_eq_	
S1	0.75328 (5)	0.70391 (3)	0.56623 (4)	0.02440 (13)	
O1	0.71400 (17)	0.45258 (11)	0.34210 (15)	0.0396 (3)	
O2	0.87198 (17)	0.40764 (11)	0.57869 (13)	0.0382 (3)	
H2B	0.8839	0.4807	0.5877	0.057*	
O3	0.90270 (15)	0.63375 (11)	0.64101 (13)	0.0340 (3)	
O4	0.59511 (16)	0.64255 (12)	0.54911 (14)	0.0391 (3)	
O5	0.75643 (18)	0.75578 (12)	0.42787 (13)	0.0395 (3)	
O6	0.76089 (19)	0.81232 (11)	0.66597 (13)	0.0414 (4)	
H1	0.7597	0.7975	0.7581	0.062*	
N1	0.60598 (19)	0.10546 (14)	0.25572 (17)	0.0366 (4)	
H1B	0.5361	0.0840	0.1701	0.044*	
C4	0.75053 (19)	0.25493 (14)	0.41590 (17)	0.0259 (3)	
C5	0.6393 (2)	0.21802 (15)	0.27984 (19)	0.0312 (4)	
H5A	0.5877	0.2715	0.2050	0.037*	
C3	0.8265 (2)	0.17308 (15)	0.52470 (19)	0.0334 (4)	
H3A	0.9031	0.1960	0.6169	0.040*	
C2	0.7879 (3)	0.05679 (16)	0.4957 (2)	0.0422 (4)	
H2A	0.8372	0.0013	0.5685	0.051*	
C6	0.7770 (2)	0.38299 (15)	0.43965 (18)	0.0282 (3)	
C1	0.6761 (3)	0.02450 (16)	0.3583 (2)	0.0414 (4)	
H1A	0.6494	−0.0532	0.3370	0.050*	

### Atomic displacement parameters (Å^2^)

**Table:**

	*U*^11^	*U*^22^	*U*^33^	*U*^12^	*U*^13^	*U*^23^
S1	0.0327 (2)	0.0247 (2)	0.01610 (19)	0.00166 (15)	0.00847 (15)	−0.00001 (13)
O1	0.0447 (7)	0.0301 (7)	0.0398 (7)	0.0039 (6)	0.0083 (6)	0.0078 (5)
O2	0.0476 (7)	0.0270 (6)	0.0332 (7)	−0.0027 (5)	0.0044 (5)	−0.0032 (5)
O3	0.0314 (6)	0.0327 (6)	0.0306 (6)	0.0016 (5)	0.0007 (5)	−0.0031 (5)
O4	0.0306 (6)	0.0493 (8)	0.0370 (7)	−0.0028 (6)	0.0104 (5)	0.0001 (6)
O5	0.0642 (9)	0.0384 (7)	0.0199 (6)	0.0022 (6)	0.0195 (6)	0.0021 (5)
O6	0.0776 (10)	0.0271 (6)	0.0241 (6)	0.0051 (6)	0.0230 (6)	−0.0019 (5)
N1	0.0338 (8)	0.0376 (8)	0.0334 (8)	−0.0030 (6)	0.0045 (6)	−0.0106 (6)
C4	0.0231 (7)	0.0274 (8)	0.0265 (8)	0.0008 (6)	0.0073 (6)	−0.0005 (6)
C5	0.0295 (8)	0.0336 (9)	0.0274 (8)	0.0029 (7)	0.0051 (6)	0.0002 (7)
C3	0.0338 (9)	0.0312 (9)	0.0291 (8)	0.0010 (7)	0.0025 (7)	0.0018 (7)
C2	0.0512 (11)	0.0281 (9)	0.0417 (11)	0.0031 (8)	0.0079 (8)	0.0069 (8)
C6	0.0251 (7)	0.0280 (8)	0.0309 (8)	0.0007 (6)	0.0086 (6)	0.0016 (6)
C1	0.0461 (11)	0.0268 (9)	0.0501 (11)	−0.0043 (8)	0.0145 (9)	−0.0059 (8)

### Geometric parameters (Å, °)

**Table:**

S1—O4	1.4478 (13)	N1—H1B	0.8600
S1—O5	1.4483 (12)	C4—C5	1.377 (2)
S1—O3	1.4493 (13)	C4—C3	1.385 (2)
S1—O6	1.5565 (12)	C4—C6	1.500 (2)
O1—C6	1.204 (2)	C5—H5A	0.9300
O2—C6	1.320 (2)	C3—C2	1.386 (3)
O2—H2B	0.8501	C3—H3A	0.9300
O6—H1	0.8921	C2—C1	1.373 (3)
N1—C5	1.332 (2)	C2—H2A	0.9300
N1—C1	1.334 (2)	C1—H1A	0.9300
			
O4—S1—O5	112.85 (8)	N1—C5—H5A	120.1
O4—S1—O3	111.86 (8)	C4—C5—H5A	120.1
O5—S1—O3	113.83 (8)	C4—C3—C2	119.75 (16)
O4—S1—O6	108.30 (8)	C4—C3—H3A	120.1
O5—S1—O6	101.94 (7)	C2—C3—H3A	120.1
O3—S1—O6	107.28 (8)	C1—C2—C3	119.25 (17)
C6—O2—H2B	108.9	C1—C2—H2A	120.4
S1—O6—H1	115.3	C3—C2—H2A	120.4
C5—N1—C1	122.94 (16)	O1—C6—O2	125.66 (16)
C5—N1—H1B	118.5	O1—C6—C4	122.57 (15)
C1—N1—H1B	118.5	O2—C6—C4	111.75 (14)
C5—C4—C3	118.73 (16)	N1—C1—C2	119.48 (17)
C5—C4—C6	117.62 (15)	N1—C1—H1A	120.3
C3—C4—C6	123.61 (15)	C2—C1—H1A	120.3
N1—C5—C4	119.85 (16)		

### Hydrogen-bond geometry (Å, °)

**Table:**

*D*—H···*A*	*D*—H	H···*A*	*D*···*A*	*D*—H···*A*
O2—H2B···O3	0.85	1.83	2.6697 (18)	169
O6—H1···O5^i^	0.89	1.73	2.6129 (17)	170
N1—H1B···O4^ii^	0.86	2.11	2.843 (2)	143

Symmetry codes: (i) *x*, −*y*+3/2, *z*+1/2; (ii) −*x*+1, *y*−1/2, −*z*+1/2.

## References

[bb1] Athimoolam, S. & Rajaram, R. K. (2005). *Acta Cryst.* E**61**, o2764–o2767.

[bb2] Czupiński, O., Bator, G., Ciunik, Z., Jakubas, R., Medycki, W. & Świergiel, J. (2002). * J. Phys. Condens. Matter*, **14**, 8497–8512.

[bb3] Katrusiak, A. & Szafrański, M. (1999). *Phys. Rev. Lett.* **82**, 576–579.

[bb4] Katrusiak, A. & Szafrański, M. (2006). *J. Am. Chem. Soc.* **128**, 15775–15785. 10.1021/ja0650192 17147387

[bb5] Rigaku (2005). *CrystalClear* Rigaku Corporation, Tokyo, Japan.

[bb6] Sheldrick, G. M. (2008). *Acta Cryst.* A**64**, 112–122. 10.1107/S0108767307043930 18156677

